# Association of metformin use with asthma development and adverse outcomes: A systematic review and meta-analysis

**DOI:** 10.1097/MD.0000000000039785

**Published:** 2024-10-04

**Authors:** Rui Rao, Juan Mei, Hudie Chen, Chuanjing Yang

**Affiliations:** a Second Affiliated Hospital of Guizhou University of Traditional Chinese Medicine, Guiyang, Guizhou, China; b Guizhou University of Traditional Chinese Medicine, Guiyang, Guizhou, China.

**Keywords:** asthma, diabetes, meta-analysis, metformin, systematic review

## Abstract

**Background:**

Asthma and diabetes are prevalent chronic diseases affecting a significant population globally. Research has suggested that metformin, a commonly used medication for diabetes management, may also have beneficial effects in enhancing asthma outcomes. Considering the comorbidity of asthma and diabetes, a comprehensive analysis was performed to investigate the efficacy of metformin in reducing adverse outcomes of asthma patients with diabetes.

**Methods:**

To gather relevant data, we conducted a systematic search of the PubMed, Embase, and CENTRAL databases for observational studies published prior to September 2023. We specifically looked for studies involving individuals diagnosed with both asthma and diabetes, comparing the incidence and severity of asthma exacerbations in metformin users versus nonusers. The inclusion criteria encompassed studies that recruited participants aged 18 years and older. The primary outcome of interest was the risk of newly developing asthma, while secondary outcomes included the adjusted risk of asthma-induced exacerbations, emergency room visits, and hospitalizations. All data analyses and visualizations were performed using the R programming language.

**Results:**

We identified and included 7 studies involving a total of 1,176,398 patients in our analysis. The pooled effect size indicated a potential reduction in the incidence of newly developed asthma among patients with type 2 diabetes who used metformin, although this finding did not reach statistical significance. Similar conclusions have also been observed in other outcomes, such as exacerbation, asthma-related emergency department visits, risk of systemic corticosteroid prescription. The only positive outcome is that the use of metformin can reduce the chance of patients being hospitalized due to asthma.

**Conclusion:**

In most outcome indicators, it cannot be assumed that the use of metformin can reduce asthma-related adverse events. However, the conclusion is not so certain, and longer observation and more evidence are still required. Metformin still shows some potential in the intervention of respiratory diseases.

## 1. Introduction

Asthma, a chronic inflammatory disorder of the small airways, is characterized by recurrent symptoms encompassing wheezing, coughing, chest constriction, and dyspnea.^[1]^ The global burden of this condition is substantial, with over 300 million individuals affected,^[2]^ and it was responsible for an estimated 461,000 fatalities in 2019.^[3]^ In particular, China harbors a significant proportion of this disease burden, with over 40 million affected individuals, and the mortality rates are particularly high in developing nations.^[4]^ Despite the absence of a definitive cure for asthma, disease management and control are feasible.^[3]^ Asthma-related morbidity and mortality can be effectively diminished through the implementation of pharmacological interventions utilizing controller and reliever medications.^[5,6]^ The application of single maintenance and reliever therapy is a viable strategy for mitigating the risk of asthma exacerbations.^[6]^ Furthermore, novel monoclonal antibodies have emerged that target conventional medications, although their efficacy and safety profiles remain to be substantiated, and their exorbitant costs often pose a barrier to access.^[7,8]^ Consequently, there is a pressing requirement for cost-effective and efficacious adjunctive therapies.

Type 2 diabetes (T2D), a constellation of metabolic disorders primarily typified by hyperglycemia, often co-occurs in adult asthmatic patients.^[9]^ Recent epidemiologic evidence suggests that metformin, the cornerstone of pharmacotherapy for T2D, might diminish the risk of asthma exacerbations.^[9–12]^ Nonetheless, the relationship between metformin use and asthma control is contentious, with reports indicating both salutary and deleterious effects. There are also indications that metformin usage is associated with elevated risks of asthma exacerbation and hospitalization.^[13]^ There was a meta-analysis that briefly evaluated this issue, but the data was not very complete at that time.^[14]^ Accordingly, this paper endeavors to address 2 research queries: firstly, the potential of metformin to reduce asthma incidence in T2D patients, and secondly, the safety of metformin use in T2D patients with concomitant asthma for the reduction of adverse outcomes. This investigation may provide valuable insights into the potential utility of metformin as adjunctive therapy for asthma management in patients with coexisting T2D.

## 2. Methods

### 2.1. Protocol and registration

The methodology employed in this systematic review has been duly registered on the internationally recognized platform for registered systematic review and meta-analysis protocols, namely the International Platform of Registered Systematic Review and Meta-Analysis Protocols (INPLASY, https://inplasy.com/). Furthermore, the reporting of the study conforms to the PRISMA^[15]^ (Preferred Reporting Items for Systematic Reviews and Meta-Analyses) guidelines, which serve as a standardized framework for transparent and comprehensive reporting of systematic reviews and meta-analyses in the field of clinical research. The registered DOI number of this review were recorded as INPLASY202340005 and 10.37766/inplasy2023.4.0005, respectively. The data utilized in this research paper have been derived exclusively from previously published scholarly sources, thereby negating the necessity for ethical approval.

### 2.2. Search strategy

A thorough literature search was conducted via electronic databases PubMed, Embase, and Cochrane Central Register of Controlled Trials (CENTRAL) using the search strategy “(asthma[Title/Abstract] AND metformin[Title/Abstract]).” The search criteria were in accordance with the Cochrane Handbook guidelines, as established by JM and RR. Date limitations were set to retrieve original studies published prior to Sep 2023. Additionally, retrieved articles and relevant reviews were examined for additional sources by HC.

### 2.3. Study selection

The study’s inclusions criteria were delineated as follows: 1 – utilization of observational studies; 2 – participants who were over 18 years old; 3 – examined the utilization of metformin for individuals afflicted with both asthma and diabetes; 4 – odds ratios (ORs) or Hazard ratios (HRs) were compared between metformin users and nonusers related to the development or aggravation of asthma. Conversely, the study’s exclusion criteria were established in the following manner: 1 – not related to cell or animal research; 2 – not related to case reports, letters to the editor or reviews; 3 – possible duplication of publications. Both JM and RR independently screened the titles and abstracts, and in case of dissension, a third investigator (CY) was consulted by the authors for a final decision.

### 2.4. Data extraction and collection

The present study involved extraction of data from selected research articles conducted by JM and HC. The extracted data encompassed key information such as author name, year of publication, study design, geographical location, age and gender of both metformin users and nonusers, exclusion and inclusion criteria, follow-up duration, outcomes, and statistical approaches. A comprehensive approach was used which was guided by Preferred Reporting Items for Systematic Reviews and Meta-Analyses^[15]^ (PRISMA) guidelines to ensure completeness, clarity, and transparency in the reporting of findings. Moreover, the Newcastle-Ottawa Scale^[16]^ was employed to assess quality aspects of individual studies reviewed.

### 2.5. Outcomes

The principal measurements employed in the present study included the adjusted probability of developing asthma in the presence of T2D, and the likelihood of experiencing asthma exacerbation in the existence of concurrent T2D and asthma. Additionally, the secondary measurements involved the adjusted likelihood of hospitalization pertaining to asthma, emergency room visits, prescription of systemic corticosteroids, and mortality.

### 2.6. Statistical analysis

The dataset underwent rigorous screening utilizing the Microsoft Office 365. Subsequently, the statistical calculations, data visualization, sensitivity analysis, and examination for publication bias were performed using the R language. The estimation of heterogeneity was carried out using both the *I*^2^ and χ^2^ tests. We would employ a random-effects model for the ensuing meta-analysis. The HRs for dichotomous variables were calculated accordingly. If the inclusion criteria for the literature are met, a sensitivity and subgroup analysis will be conducted to identify the sources of heterogeneity. The confidence interval (CI) for the statistical measure is set at 95%. If there is sufficient literature included, publication bias will be evaluated using a funnel plot.

## 3. Results

### 3.1. Overall description and risk of bias

**Figure 1.**
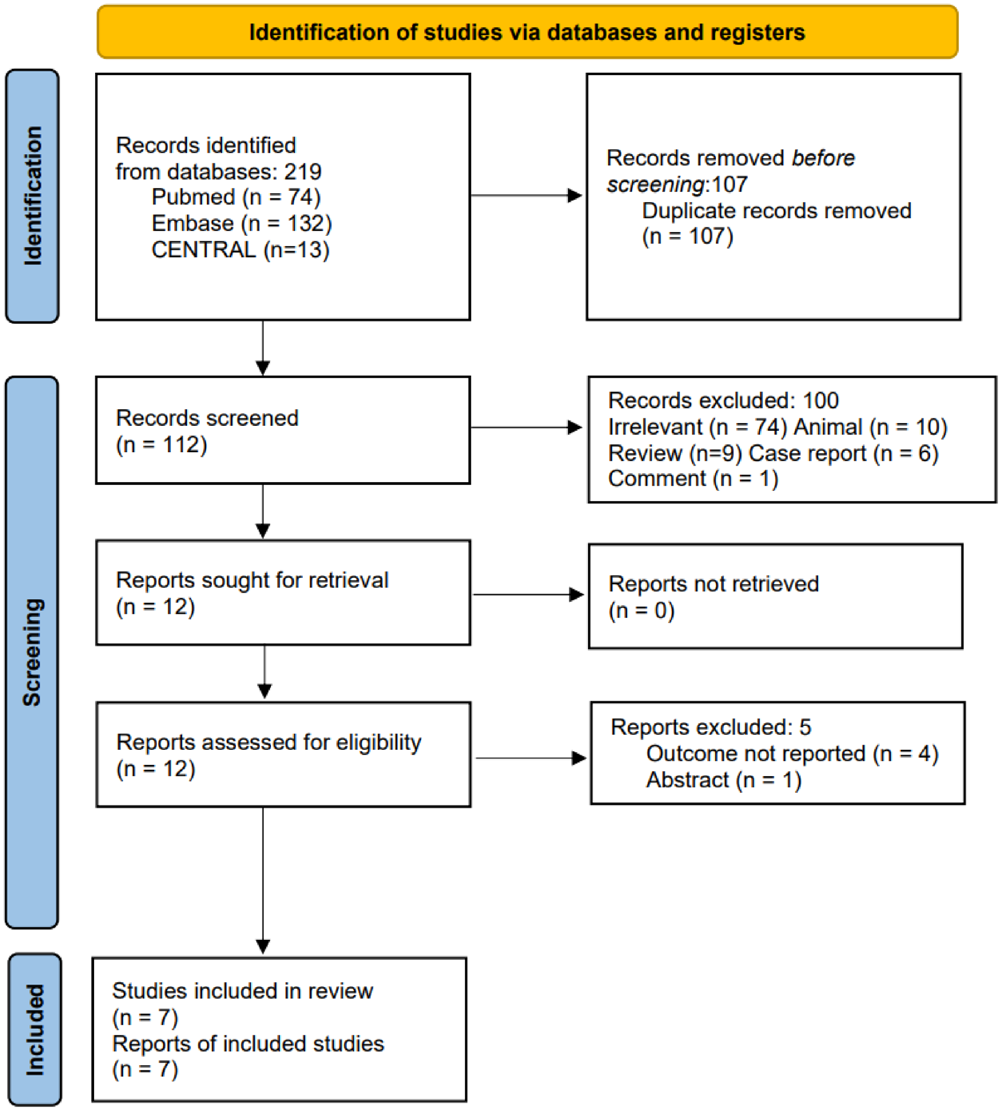
Flow diagram of the study selection.

Through implementation of the aforementioned search strategy, a cumulative total of 112 scholarly articles were identified subsequent to removal of duplicate records. Following a meticulous assessment of the titles and abstracts, twelve records were retrieved in their entirety, of which 7 were deemed suitable for inclusion in our analysis, comprising a combined participant population of 1,176,398 individuals. A graphical representation outlining the selection process can be observed in Figure 1. Collectively, these investigations encompassed 7 observational studies, specifically studies,^[9–12,17–19]^ and their characteristics are documented in Table 1. The chosen trials were also assessed to be of high quality, with the risk of bias evaluated employing the Newcastle-Ottawa Scale as detailed in Table 2.

**Table 1:** Characteristics of the eligible trials and their participants.

First author (yr)	Region	Design	Sample size		Gender		Age		Criteria of Inclusion and Exclusion	Follow-Up (years)	Outcomes	Statistical methods	Adjustments
			Exposure	Control	Exposure	Control	Exposure	Control					
Rayner 2019^[9]^	UK	RC	29,222	865,424	16,811/12,411	396,365/469,059	69.83 ± 12.3	48.54 ± 18.3	Inclusion: All registered patients aged over 18. Exclusion: A baseline diagnosis of asthma, COPD or T1DM.	7.89	a	CPHR	Age, gender, BMI, smoking status and IMD score
Li 2016^[11]^	Taiwan	RC	444	888	176/268	352/536	64 ± 10.1	64 ± 10.1	Inclusion Criteria: 1. Aged ≥ 18 years with concurrent asthma and diabetes. 2. Who have had at least 1 inpatient or 2 outpatient diagnoses of asthma and diabetes during the enrollment period. 3. Who had at least 1 prescription for asthma and diabetes medication during the enrollment period. 4.Matched patient’s the date of the asthma and diabetes diagnosis must be earlier than the index date. Exclusion Criteria: 1. Individuals who had a prescription for metformin within the 1-year period prior to the index date will be excluded from the study. 2. Patients who have been diagnosed with chronic obstructive pulmonary disease (COPD), any respiratory tract cancer, or bronchiectasis during the pre-index period will be excluded from the analysis. 3. Patients who have experienced an asthma-related hospitalization or emergency room visit during the pre-index period will be excluded from the study. 4. Patients with missing or invalid demographic information, including age, gender, diagnosis codes, medication prescriptions, and enrollment records, will also be excluded from the analysis.	3	b, c, and d	LR	Age, gender, region, Charlson comorbidity index, duration of asthma, medications for asthma and medication for diabetes.
Eskin 2019^[17]^	Canada	RC	130,725	4576	73,680/57,045	2307/2269	55 ± 13	60 ± 16	Inclusion: 1. At least 30 years of age. 2. New oral antihyperglycemic medication users. Exclusion: Women using metformin as a single oral antihyperglycemic medication for polycystic ovary syndrome.	3.4	a	CPHR	Age sex, Elixhauser comorbidity score and other antidiabetes medications use, anticoagulants, beta blockers, calcium channel blockers, diuretics, nitrates use and the number of hospitalization per year prior to cohort entry, healthy user markers (ACEi and statins use, flu and pneumonia vaccinations, screening tests, accident events, motor vehicle accidents
Wu 2019^[12]^	USA	RC	11,960	11,960	4066/7894	4058/7902	51.9 ± 9.3	51.9 ± 9.9	Inclusion Criteria: 1. The study includes adult participants aged 18 or older who have been diagnosed with both asthma and diabetes. 2. Furthermore, they must have had at least 2 outpatient codes or 1 inpatient code that indicate the presence of both conditions during the enrollment period. 3. Additionally, qualifying outpatient claims should be documented within a 1-year timeframe. Exclusion Criteria: 1. Individuals with a diagnosis of chronic obstructive pulmonary disease, bronchiectasis, or interstitial lung disease are excluded from the study. 2. Moreover, individuals with contraindications for the use of metformin, those with type 1 diabetes, and those with a rheumatologic condition that may require systemic corticosteroids for symptoms unrelated to asthma are also excluded from the study.	0.84	b, c, d, and e	CPHR	Age, medications, Charlson comorbidity index, asthma severity, region, months of eligible enrollment.
Wu 2021	USA	RC	861	888	223/638	238/650	53.3 ± 13.6	55.1 ± 14.3	Inclusion: 1. Adult (age > 18 years) patients with asthma on the basis of 2 separate outpatient or 1 inpatient International Classification of Diseases diagnostic code for asthma recorded. 2. Patients who had hemoglobin A1c (HbA1c) testing with an HbA1c value of greater than or equal to 6.5 or taking diabetes medications. Exclusion: 1. Patients with chronic kidney disease and type 1 diabetes. 2. Individuals with chronic lung disease other than asthma or an active order for a systemic corticosteroid during their index date.	3.2	c, d, and e	CPHR	Age, sex, race, smoking status, BMI, Charlson comorbidity index, insurance type, and use of thiazolidinedione or GLP-1 medications.
Chen 2017^[10]^	Taiwan	CC	1982#	1982#	965/1017	965/1017	61.7 ± 13.9#	61.6 ± 13.8#	Inclusion: Diabetes Exclusion: 1. Gender was unknown. 2. Diagnosis of asthma before their first diabetes diagnosis.	NA	a	LR	Age, gender and obesity.
Yen 2022^[13]^	Taiwan	RC	57,743	57,743	28,626/29,117	28,579/29,164	57.0 (12.89)		Inclusion: 1. Newly diagnosed T2D Exclusion: 1. Missing data on age or gender. 2. Age below 20 or above 80 years. 3. Diagnosis of type 1 diabetes, asthma, hepatic failure, or received dialysis. 4. Previous diagnosis of T2D before 1 January 2000	6.67/4.15	a	CPHR	Sex, age, obesity, smoking, comorbidities, CCI and DCSI scores, OHA, insulin, statin, aspirin, immunosuppressants, influenza vaccination, adult health examination, HbA1C > 2 times per year.

Outcomes: a, new incidence of asthma; b, exacerbation; c, emergency room visit; d, hospitalization; e, systemic steroid prescription.

CC = case control, CPHR = Cox Proportional Hazards Regression, LR = logical regression, OHA = oral hypoglycemic agents, RC = retrospective cohort.

**Table 2:** Assessment of bias risks of the included publications.

	SELECTION				COMPARABILITY	Outcome		
	1) Representativeness of the exposed cohort	2) Selection of the non-exposed cohort	3) Ascertainment of exposure	4) Demonstration that outcome of interest was not present at start of study	1) Comparability of cohorts on the basis of the design or analysis	1) Assessment of outcome	2) Was follow-up long enough for outcomes to occur	3) Adequacy of follow-up of cohorts
Li 2016^[11]^	1	1	1	1	1	1	1	1
Wu 2019^[12]^	1	1	1	1	1	1	1	1
Wu 2021	1	1	1	1	1	1	1	1
Rayner 2019^[9]^	1	1	1	1	1	1	1	1
Eskin 2019^[17]^	1	1	1	1	1	1	1	1
Mendy 2019^[18]^	1	1	1	1	1	1	1	1
Chen 2017^[10]^	1	1	1	1	1	1	1	1

### 3.2. Primary outcome

**Figure 2.**
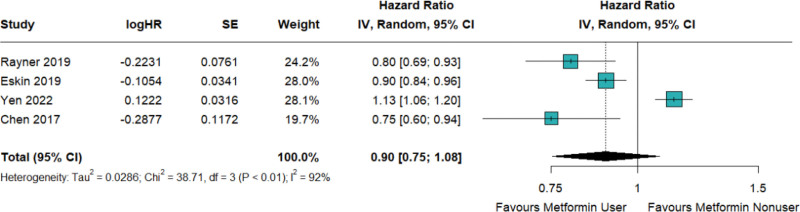
Metformin demonstrated a reduction in the incidence of newly developed asthma in patients with T2D, not achieving statistical significance.

In relation to individuals diagnosed with T2D, 4 publications were identified that reported on the risk of newly developed asthma. The findings of the meta-analysis suggest a decreased likelihood of asthma incidence in the studied population following treatment with metformin (HR = 0.90; 95% CI: 0.75–1.08; *I*^2^ = 92%; Fig. 2). However, there was a significant degree of heterogeneity observed across the included studies. Sensitivity analysis shows that 1 publication significantly skewed the results.^[13]^ Exclusion of the particular article^[13]^ yields a statistically significant result supporting the protective effect of metformin against asthma development (HR = 0.84; 95% CI: 0.76–0.94; Figure S1, Supplemental Digital Content, http://links.lww.com/MD/N635).

### 3.3. Secondary outcomes

**Figure 3.**
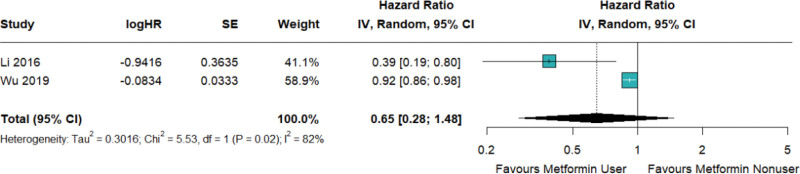
Metformin decreased the risk of asthma exacerbation in patients with type2 diabetes without reaching the statistical significance.

The first 1 of the secondary outcomes was the risk of asthma exacerbation in patients who have both diabetes and asthma. The data presented a high level of heterogeneity, and it was observed that the effect of metformin was not significant with a HR of 0.65 and a 95% CI of 0.28 to 1.48, as illustrated in Figure 3 and Figure S2, Supplemental Digital Content, http://links.lww.com/MD/N636.

**Figure 4.**
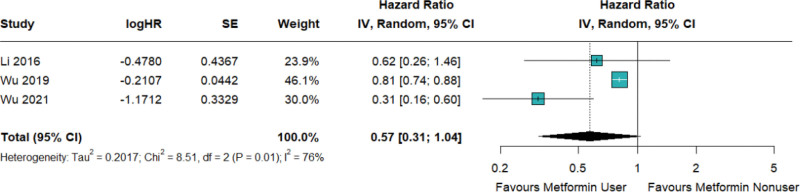
Metformin decreased the risk of asthma-related emergency room visits in patients with concurrent asthma and diabetes, but this decrease was not statistically significant.

In 3 observational studies examining the relationship between metformin and asthma-related emergency room visits, it was observed that metformin use did not decrease the risk of asthma-related emergency room visits with significance (HR = 0.57; 95% CI: 0.31–1.04; *I*^2^ = 76%; Fig. 4; Figure S3, Supplemental Digital Content, http://links.lww.com/MD/N637).

**Figure 5.**
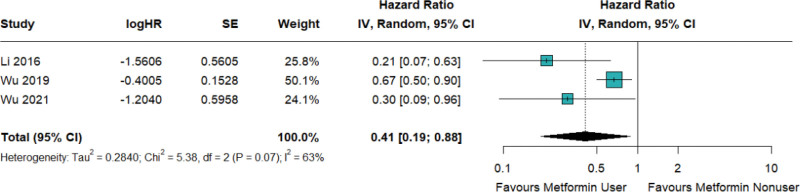
Metformin decreased the risk of asthma-related hospitalization in patients with concurrent asthma and diabetes.

The present study findings indicate that the utilization of metformin is associated with a reduction in the likelihood of asthma-related hospitalization among individuals who have concurrent diagnoses of diabetes and asthma (HR = 0.41; 95% CI: 0.19–0.88; *I*^2^ = 91%; Fig. 5; Figure S4, Supplemental Digital Content, http://links.lww.com/MD/N638).

**Figure 6.**
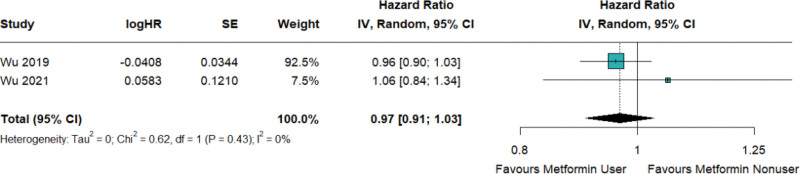
Metformin decreased the risk of systemic corticosteroid use in patients with T2D and asthma.

Only 2 study reported the risk of systemic corticosteroid prescription as an outcome. The conclusion of this study demonstrates that metformin does not significantly reduce the probability of using systemic corticosteroids (HR = 0.97; 95% CI: 0.91–1.03; Fig. 6).

## 4. Discussion

In this meta-analysis focusing on adults with concurrent asthma and diabetes, our findings suggest an inverse association between the use of metformin and the occurrence of asthma. Furthermore, we observed a decreased risk of asthma attack, asthma-induced visits to the emergency department, hospitalizations due to asthma, and the need for systemic corticosteroid therapy among individuals treated with metformin. However, it is unfortunate that, apart from the risk of hospitalization, the remaining indicators do not yield positive statistical results.

When examining the studies individually, it was found that metformin was associated with a reduced risk of the 3 outcomes. Through sensitivity analysis, we noticed that 1 author’s conclusions yielded opposite trends compared to the other authors.^[13]^ Furthermore, in the most recent literature published in 2024, it was reported that following the prescription of metformin, there was a notable decline in the rates of asthma exacerbations. However, to ascertain whether this reduction can be attributed to the use of metformin, it is essential to conduct a study with a more extensive patient sample size, including those who have experienced exacerbations and others with asthma who have not been prescribed metformin.^[20]^ Although this particular study was not incorporated into the meta-analysis due to the outcome measures utilized, it remains imperative to introduce and emphasize it within the discourse.

We also paid attention to the effects of metformin on other respiratory diseases, such as COPD, which was found to contradict the findings of the author^[21]^ and other literature.^[22–24]^ The emergence of such heterogeneity seemed quite unusual. Therefore, we screened some possible factors that could lead to this heterogeneity, such as age, ethnicity, inclusion/exclusion criteria, statistical methods, but found no specific abnormality. Due to limited data, we could not determine what factors caused the heterogeneity. Nevertheless, these findings indicate a prospective utility of metformin in reducing the likelihood of exacerbations of asthma along with other respiratory conditions.

Metformin, a commonly prescribed medication for diabetes management, has been demonstrated to be linked with a reduced risk of mortality associated with respiratory disease. In contrast, other antidiabetic medications have not shown a comparable effect in reducing this risk.^[18]^ Numerous studies have reported the potential benefits of metformin use in reducing the risk of asthma development among individuals with diabetes.^[9,10,25]^

The mechanism by which metformin may potentially treat asthma is not well understood and is still an area of active research. However, there are several proposed mechanisms that have been suggested based on preclinical and clinical studies.

One possible mechanism is through the activation of AMP-activated protein kinase (AMPK). AMPK is a cellular energy sensor that is involved in regulating glucose metabolism, lipid metabolism, and inflammation. Studies have shown that metformin activates AMPK in various cell types, including lung epithelial cells and immune cells, which may help to reduce airway inflammation^[26]^ and improve lung function^[27]^ in individuals with asthma.

Metformin potential in treating asthma may also involve immunomodulation, specifically in regulating the balance of Treg/Th17 cells, which plays a pivotal role in asthma pathogenesis. A study on mice showed that metformin increased the Treg/Th17 ratio,^[28]^ which suggests that this may be another avenue through which metformin exerts its anti-asthma effects.

The third potential mechanism is through the regulation of gut microbiota. There is increasing evidence to suggest that gut dysbiosis, or an imbalance in the gut microbiota, may play a role in the development and progression of asthma. Metformin has been shown to alter the composition of gut microbiota in animal models and humans, which may potentially lead to a reduction in airway inflammation and improved asthma symptoms. These proposed mechanisms are still being investigated and further research is needed to fully understand how metformin may potentially treat asthma. Our research group is also actively preparing experiments to try to uncover some of the potential mechanisms of metformin in respiratory system diseases.

There are several limitations that must be acknowledged. Firstly, the number of studies included in this meta-analysis was limited to 7, which is a relatively small sample size. Consequently, we were not able to carry out subgroup analyses or evaluate the potential for publication bias. Moreover, owing to the dearth of research studies that adhere to rigorous methodological standards, there is a need for additional empirical investigations in order to substantiate the accuracy and reliability of the findings derived from the present review.

## 5. Conclusion

Drawing from the available literature, establishing the efficacy of metformin as an intervention approach for asthma is a challenging task. While certain studies indicate to the possible reduction of adverse asthma-related occurrences upon metformin administration, there exist contrasting reports which warrant comprehensive scrutiny. The presence of such observations implies a plausible involvement of metformin in the respiratory system. To assert metformin therapeutic viability, not only are controlled trials warranted, but also a need to unravel the underlying mechanisms of these effects.

## Acknowledgments

The data, which provide evidence for the conclusions drawn in this study, are accessible in publicly available databases.

DOI: 10.1111/crj.13011, 10.1111/resp.12818, 10.1016/j.jcjd.2018.12.001, 10.1513/AnnalsATS.201812-897OC, 10.1016/j.jaip.2021.07.007, 10.1080/02770903.2017.1283698, 10.3390/ijerph19138211.

## Author contributions

**Data curation:** Rui Rao, Juan Mei, Hudie Chen.

**Writing – original draft:** Rui Rao.

**Writing – review & editing:** Chuanjing Yang.

## Supplementary Material


