# Supplementary figures and images for: Association of metformin use with asthma development and adverse outcomes: A systematic review and meta-analysis
Source: Medicine (Baltimore). 2024 Oct 4;103(40):e39785. doi: 10.1097/MD.0000000000039785 (PMC11460891; doi:10.1097/MD.0000000000039785)

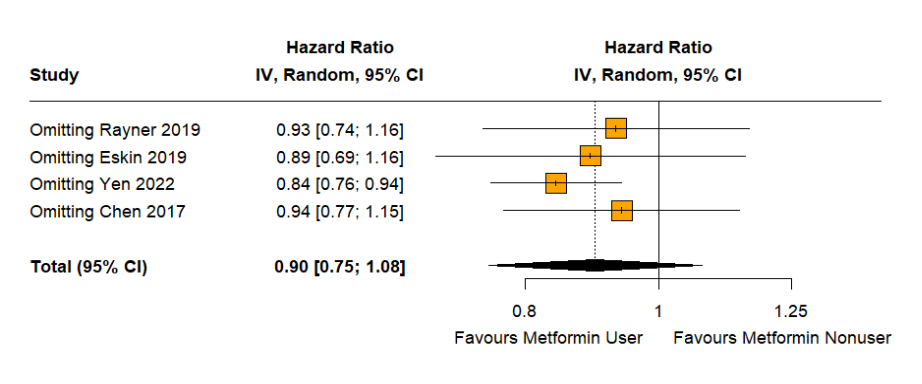

Supplement: Supplementary file 1 [file medi-103-e39785-s001.docx]

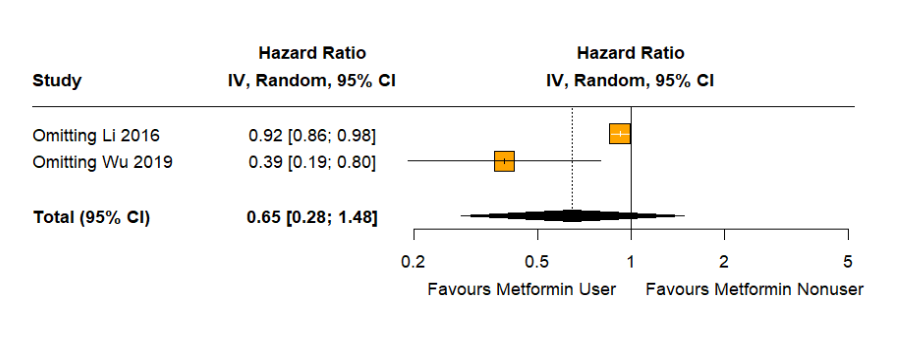

Supplement: Supplementary file 2 [file medi-103-e39785-s002.docx]

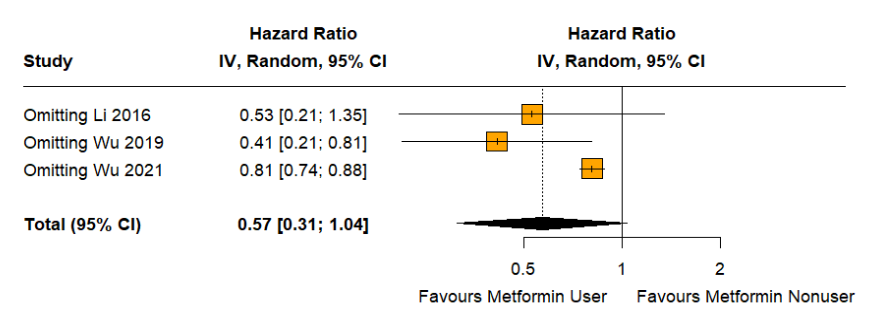

Supplement: Supplementary file 3 [file medi-103-e39785-s003.docx]

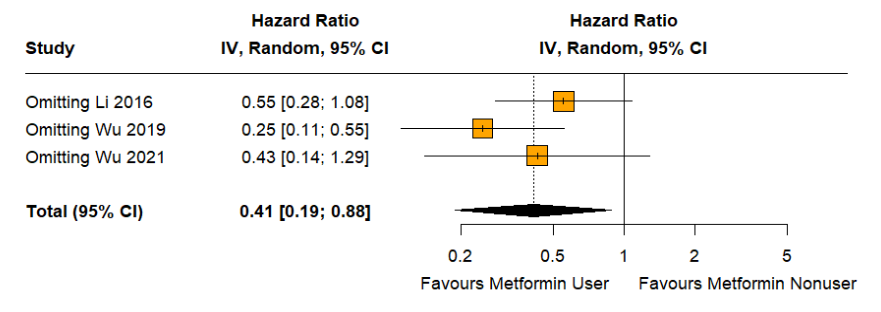

Supplement: Supplementary file 4 [file medi-103-e39785-s004.docx]
